# Experimental Tests and Numerical Simulations on the Ballistic Impact Response of a Highly Inhomogeneous Aluminium Foam

**DOI:** 10.3390/ma15134651

**Published:** 2022-07-01

**Authors:** Kristoffer A. Brekken, Ole Vestrum, Sumita Dey, Aase Reyes, Tore Børvik

**Affiliations:** 1 Structural Impact Laboratory (SIMLab), Department of Structural Engineering, NTNU—Norwegian University of Science and Technology, 7491 Trondheim, Norway; tore.borvik@ntnu.no; 2 Centre for Advanced Structural Analysis (CASA), NTNU—Norwegian University of Science and Technology, 7491 Trondheim, Norway; 3 Research and Development Department, Norwegian Defence Estates Agency, 0103 Oslo, Norway; ole.vestrum@forsvarsbygg.no (O.V.); sumita.dey@forsvarsbygg.no (S.D.); 4 Department of Built Environment and Energy Technology, Oslo Metropolitan University, 0130 Oslo, Norway; aase.reyes@oslomet.no

**Keywords:** sandwich structures, cellular materials, XRMCT, material tests, impact tests, LS-DYNA simulations

## Abstract

A sandwich structure is a composite material consisting of thin skins encapsulating a cellular core. Such structures have proven to be excellent energy absorbents and are frequently found in various types of protection. Even so, few studies exist in the open literature on the response of the core material itself under extreme loadings such as blast and impact. Since a blast load is usually accompanied by numerous fragments, it is important to understand and be able to predict the ballistic impact resistance of the often highly inhomogeneous cellular core materials in design. In this study, the ballistic impact response of an aluminium foam with a complex cell structure has been investigated both experimentally and numerically. First, an extensive material test program involving compression tests on cubic specimens loaded in the thickness direction of the foam was carried out to reveal the mechanical properties of the material. In addition, several of the specimens were scanned before testing using X-ray Micro Computed Tomography (XRMCT) to map the multi-scale topology and morphology of the material. These data were later analysed to extract density-variation plots in many different material orientations. Second, ballistic impact tests were conducted using a gas gun where rigid spheres were launched towards aluminium foam plates, and the ballistic limit velocity and curve of the foam material were established. Finally, numerical simulations of both the material tests and the ballistic impact tests were carried out using LS-DYNA and different modelling approaches based on the XRMCT data. It will be shown that, independent of the modelling strategy applied, good agreement between the experimental impact tests and the numerical predictions can be obtained. However, XRMCT data are important if the final goal is to numerically optimise and improve the behaviour of inhomogeneous foams with respect to energy absorption, thermal isolation, or similar properties.

## 1. Introduction

Sandwich structures, here defined as composite components made of thin plates encapsulating a cellular core, have traditionally been used as structural elements with high specific bending stiffness and strength, where the main purpose of the core is to separate and stabilise the outer sheets against buckling under edgewise compression, torsion, or bending [1]. However, they have also proven to be excellent energy absorbents and may be used as safety devices in, for example, façades to mitigate blast loading [2] or cars for increased crashworthiness [3,4]. The cellular core may consist of materials such as honeycombs, open- or closed-cell polymeric or metal foams, or metal hollow spheres. For ballistic applications, the combination of the face sheets and core plays a vital role in improving the impact resistance [5]. Metal foams made of aluminium have received a lot of attention due to their many positive features such as outstanding mechanical properties, heat transfer performance, and energy dissipation capacity [6]. The capacity of metal foams to undergo large strains in compression at almost constant stress without generating damaging peak stresses makes them ideal for energy-absorption devices [7]. The properties of metallic foams depend directly on those of the solid material from which they are made and their relative density, but they are also influenced by their cell topology (open/closed cell), cell size, cell shape, and density distribution or inhomogeneity [7,8], while a comprehensive review on the dynamic compressive behaviour of cellular materials was carried out by Sun and Li [9].

Due to their positive characteristics, aluminium foams find their use in a number of engineering applications, such as the core material in sandwich structures [10,11,12,13], filler material in crash boxes for improved crashworthiness [14,15], and even as a projectile to mimic shock loading from a blast load [16,17]. Aluminium foam sandwich panels (AFS) have been evaluated for many applications, such as an Ariane 5 rocket adaptor [18], solar thermal energy generation, cooking equipment, architectural panels, and blast and bullet protection [19]. Hommel et al. [20] tried to identify profitable applications for the AFS and developed a set of motivators for the use of the aluminium foam sandwich. A survey revealed that potential users of AFS need design support and recommendations on how to design with these relatively new materials [21]. Epasto et al. [22] recently evaluated aluminium foam sandwiches subjected to impact, in the development of the Metallic Foam Shell for use as a protective device against flying ballast impact damage in railway axles, and possibly in offshore structures. Several configurations, foam core densities, and impact energies were tested, and damage and densification in the panels were successfully evaluated by means of digital radiography and ultrasonic phased array (UPA). In 2010, Hou et al. [13] performed ballistic impact experiments on metallic sandwich panels with an aluminium foam core. They found that thicker skins and cores with higher thickness and density produce higher ballistic limits, and also saw a dynamic energy enhancement although neither face nor core materials exhibited evident dynamic effects.

Recently, Zhao et al. [23] studied the mechanical properties and energy absorption capabilities of aluminium foam sandwich structures subjected to low-velocity impact. In addition to validation experiments, they used Abaqus/Explicit to model the sandwich structures and used a continuum model to model the foam together with a shear fracture criterion. They found that the thickness of the face sheet, core height, and density had a significant influence on the sandwich structure’s resistance to impact. In 2016, Elnasri and Zhao [24] studied aluminium sandwich panels under impact indentation/perforation numerically. They used LS-DYNA with an isotropic hardening elastic-plastic constitutive law with damage for the aluminium sheets and a honeycomb constitutive model for the foam material. They were able to predict their experimental results from 2007 where they found a significant enhancement of the peak loads under impact loading, although the foam core and skin sheets were known to hardly be rate-sensitive [25]. Their results indicated that the increase in the piercing force of the top skin is due to the shock enhancement of the foam core [24]. Blast loading on aluminium foam panels, and the optimisation of such panels, were extensively studied by Qi et al. [26]. They used LS-DYNA for numerical analyses and found that panels with an aluminium front face and steel back face outperformed other panel configurations in terms of maximum back plate deflection. Through artificial neural networks and multi-objective design optimisations, they found that variations in blast loading should be considered in the design of such sandwich armours to achieve more robust blast-resistant performance. Ren et al. [27] tested the influence of stacking several layers of aluminium foam and found that stacking does not necessarily have any significant influence on the absorbed energy. However, adding thin sheets between the layers had a significant effect on energy absorption.

Although aluminium foam has been produced and researched for more than twenty years, it is still the object of many studies. It is believed to be useful in, for example, sacrificial claddings or energy absorbers, by means of sandwich structures. There are still qualities/properties that are not fully understood and, especially, the predictability of numerical modelling of structures subjected to ballistic impact or blast loading, or multi-axial stress states can be improved. In this respect, many authors have looked to the micromechanical modelling of foams. Morton [28] built a micromechanical model based on Laguerre–Voronoi tessellations of a polymer foam to better understand the mechanical response of foam structures subjected to complex loading conditions. Zhang et al. [29,30] successfully used a mesoscopic 3D Voronoi model to investigate forming of aluminium foam sandwich panels (AFSP) in Abaqus/explicit. They found that the Voronoi AFSP models could reflect the actual defects, shape error, and thickness variation occurring in the plastic forming of doubly curved sandwich panels [29], and that core cracking could be predicted with the use of the Ko fracture criterion [31].

To build realistic micromechanical models, X-ray tomography has been frequently used. Tomography today refers to a 3D virtual volume reconstructed from hundreds or thousands of 2D images [32]. The possibilities of creating these virtual volumes based on many different techniques seem endless, however, scientific communities are continuously working on how to use the information in modelling, e.g., mechanical behaviour. Tomography can be used to uncover the actual microstructure of a material or the architecture or morphology of a foam and similar materials. Furthermore, changes in the bulk material of a foam could also be discovered by tomography [28]. Important parameters for foams are [33]: (1) Global density and density distribution in the foam, (2) Cell size distribution, and (3) Wall thickness distribution. Some of these parameters can be obtained by conventional techniques, but for some of them, 3D data are needed and can simplify characterisation. All in all, a better representation of the microstructure (inclusions, pores, and grains) is possible with the use of tomography, and a more accurate description of what is happening in the bulk of the studied materials can be obtained [34]. Crupi et al. [35] used a 3D computed tomography system to analyse the impacted damage of the aluminium foam core, undetectable by visual inspection, in aluminium foam sandwich panels subjected to impact. In 2017 Petit et al. [36] used X-ray tomography to characterise and model the mechanical behaviour of open-cell aluminium foam by considering its microstructural features. Here, FE models of inclusions were built, and specific constitutive laws were used for the aluminium matrix and the inclusions. The presence of the inclusions resulted in local stress concentration explaining the early fracture of some struts where no specific architectural features were noticed. Recently, Vestrum et al. [37,38,39] used X-ray micro computed tomography (XRMCT) to map the density of polymer coating and build FE models of the porous morphology. The FE models were used to calibrate a constitutive material model that takes the porosity of the coating into account.

In the present study, the ballistic impact response of an aluminium foam with a complex cell structure has been investigated both experimentally and numerically. First, an extensive material test program involving compression tests on cubic specimens loaded in the thickness direction of the foam was carried out to reveal the mechanical properties of the material. In addition, several of the specimens were scanned before testing using X-ray Micro Computed Tomography (XRMCT) to map the multi-scale topology and morphology of the material. These data were later analysed to extract density-variation plots in many different material orientations. Second, ballistic impact tests were conducted using a gas gun where rigid spheres were launched towards aluminium foam plates, and the ballistic limit velocity and curve of the foam material were established. Finally, numerical simulations of both the material tests and the ballistic impact tests were carried out using LS-DYNA and different modelling approaches based on the XRMCT data. It will be shown that, independent of the modelling strategy applied, good agreement between the experimental impact tests and the numerical predictions can be obtained. However, XRMCT data are important if the final goal is to numerically optimise and improve the behaviour of inhomogeneous foams with respect to energy absorption, thermal isolation, or similar properties.

## 2. Characterisation of the Aluminium Foam Material

### 2.1. Aluminium Foam Material

The closed-cell aluminium foam applied in this study was provided by Havel Metal Foam [40]. There are various ways to produce aluminium foams, but this foam is produced by the so-called powder compaction method [41]. Here, the process starts by mixing the metallic powder with small amounts of a foaming agent (typically TiH_2_). The powder is then compacted by uniaxial compression, rod extrusion, or rolling to obtain an almost massive component with negligible porosity before it is heated in an infrared oven to melt the aluminium matrix and decompose the foaming agent. The component is consequently “foamed up”, and panels with a porous core and thin (typically less than 0.5 mm thick) external skins can be produced. These external skins are relatively, but not completely, continuous and homogeneous. Normally, such panels have a thickness between 8–25 mm. It has been reported in the literature that thicker ingots may result in a highly inhomogeneous pore distribution due to difficulties in controlling the foaming process [41,42].

**Figure 1:**
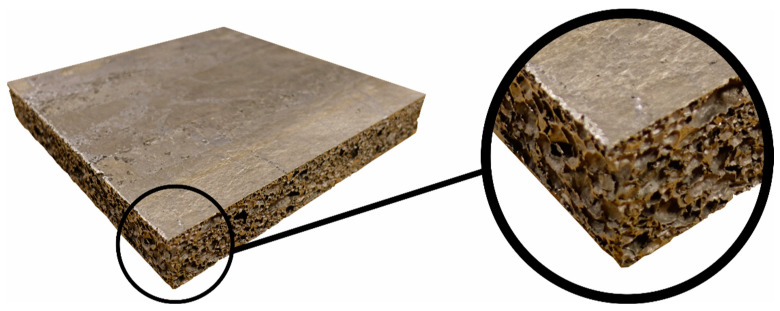
Typical aluminium foam panel applied in the ballistic impact tests, showing the external skins and the inhomogeneous porous core.

In this study, panels with in-plane dimensions of 300 mm × 300 mm (cut from a larger plate) and a nominal thickness of 50 mm are used, so an inhomogeneous pore distribution is expected. This is also illustrated in Figure 1, showing the morphology of the foam with many small and a few very large pores (with diameters of more than 1 mm) of a panel applied in the ballistic impact tests. This highly inhomogeneous pore structure is challenging for detailed numerical modelling. It is further assumed that the density of the compacted base material is 2700 kg/m^3^, i.e., the density of a standard aluminium alloy. The external skin can also be seen in the figure.

### 2.2. Mechanical Behaviour of the Aluminium Foam

Uniaxial compression tests of the aluminium foam were performed under displacement control in an Instron universal testing machine. Due to the inhomogeneous pore distribution of the applied foam, a large number (20 in total) of cubic specimens with edge lengths of 50 mm were cut from the as-received plates. The specimens included the external skins. Prior to testing, each specimen was numbered before being carefully measured and weighed. Based on these measurements, the density of the specimens was determined. As expected, the variation in density was significant, ranging from 351 kg/m^3^ to 633 kg/m^3^, with an average of 491 kg/m^3^. This gave a relative density of the foam between 0.13 and 0.23. Thus, the material can be considered a proper foam according to the definition given by Gibson and Ashby [8] since the relative density is below 0.3.

**Figure 2:**
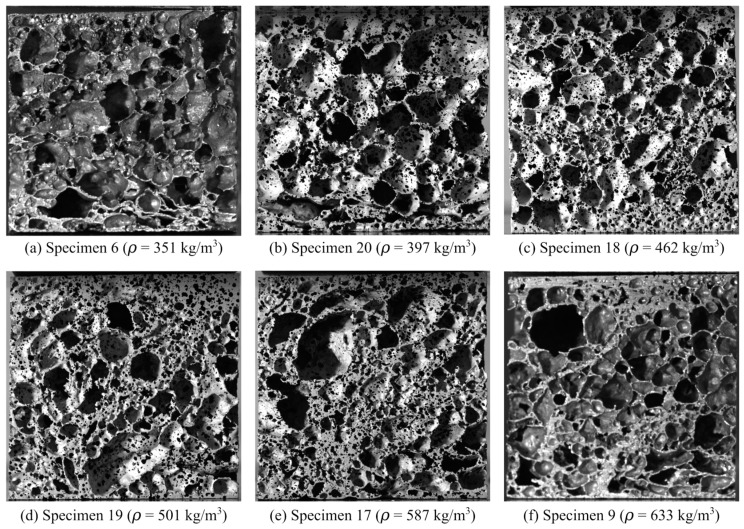
A selection of aluminium foam specimens applied in the compression tests showing a highly inhomogeneous pore distribution. The pictures are ordered so that image (**a**) shows the specimen with the lowest density while image (**f**) shows the specimen with the highest density.

The crosshead velocity of the test machine was set to 3.0 mm/min in all tests, giving an initial strain rate of 1 × 10^−3^ s^−1^ in the material, thus, the loading is considered quasi-static. During testing, the specimens were compressed between two rigid steel plates facing the external skins. The load P was measured by a 100 kN load cell attached to the actuator, while the vertical displacement w was measured both by the stroke of the actuator and edge trace of the rigid platens by means of a predefined virtual vector using the DIC-code eCorr [43]. The pictures for the DIC-analyses were provided by a Prosilica GC2450 digital camera synchronised with the load measurements. The camera was equipped with a 50 mm Nikon lens, and the image resolution in these tests was 2448 × 2050 pixels at 8-bit pixel depth. The frame rate of the camera was set to 0.5 Hz, while data from the load cell were logged at a frequency of 10 Hz. Note that only the specimens that had been CT-scanned in advance (see Section 2.3) were applied a fine-graded speckle pattern to increase the contrast in the DIC measurements. This speckle pattern is barely visible on some of the specimens in Figure 2. All tests were automatically stopped when the force approached 100 kN, which is the capacity of the load cell.

From these measurements, the engineering stress s and the engineering strain e were calculated as

(1) $$s=\frac{P}{A_{0}},\;e=\frac{w}{w_{0}}$$

where $A_{0}$ is the initial cross-section area and $w_{0}$ is the initial height of the specimen. Assuming a negligible Poisson’s ratio for the foam, i.e., $A_{0}≅A$, the true stress σ, and the true strain ε, were obtained as

(2) $$\sigma =\frac{P}{A}=\frac{P}{A_{0}},\;\varepsilon =\ln\left(e+1\right)$$

**Figure 3:**
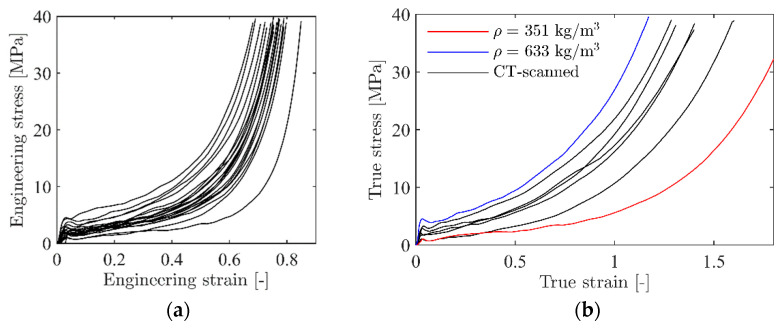
(**a**) Engineering stress-strain curves from all 20 uniaxial compression tests and (**b**) true stress-strain curves of representative tests from (**a**).

Figure 3a shows engineering stress-strain curves from all 20 uniaxial compression tests based on DIC measurements, showing the scatter in the results, while Figure 3b shows the true stress-strain curves from the CT-scanned specimens (see Section 2.3). As expected, the scatter in the stress-strain response between identical tests is significant, but all specimens display typical closed-cell foam behaviour with a linear elastic region, a plateau region with an almost linear increase in plastic strain with increasing stress, and a densification region where the cells become compacted. Mechanical properties, i.e., density ρ, elastic modulus *E*, yield stress $\sigma_{y}$, plateau stress $\sigma_{p},$ and densification strain $\varepsilon_{D},$ for some representative tests are given in Table 1. Here, E is calculated from the linear elastic region of the stress-strain curve, $\sigma_{p}$ is taken as the mean stress in the interval between 0.2 and 0.4 compressive strain, while $\sigma_{y}$ and $\varepsilon_{D}$ are obtained by a best fit to a crushable foam model from the literature (see [44] and Section 4.1 for details). As already indicated in Figure 2, specimen 6 has the lowest density and specimen 9 has the highest density, while specimen 16 has the density closest to the average density of all the tests carried out. The values in Table 1 clearly demonstrate the strong dependency of the foam’s mechanical properties on the density. When the density is increased by roughly 80% (specimen 6 versus specimen 9), the elastic modulus is more than doubled, the yield stress is increased by a factor of 4.5, and the plateau stress is more than tripled. However, the densification strain is much decreased when the density is increased. The general trends from Figure 3 and Table 1 are that the elastic modulus, the yield stress, and the plateau stress increase, while the densification strain decreases, as the density increases. It is assumed important to take these effects into account when modelling such heterogeneous foams.

**Figure 4:**
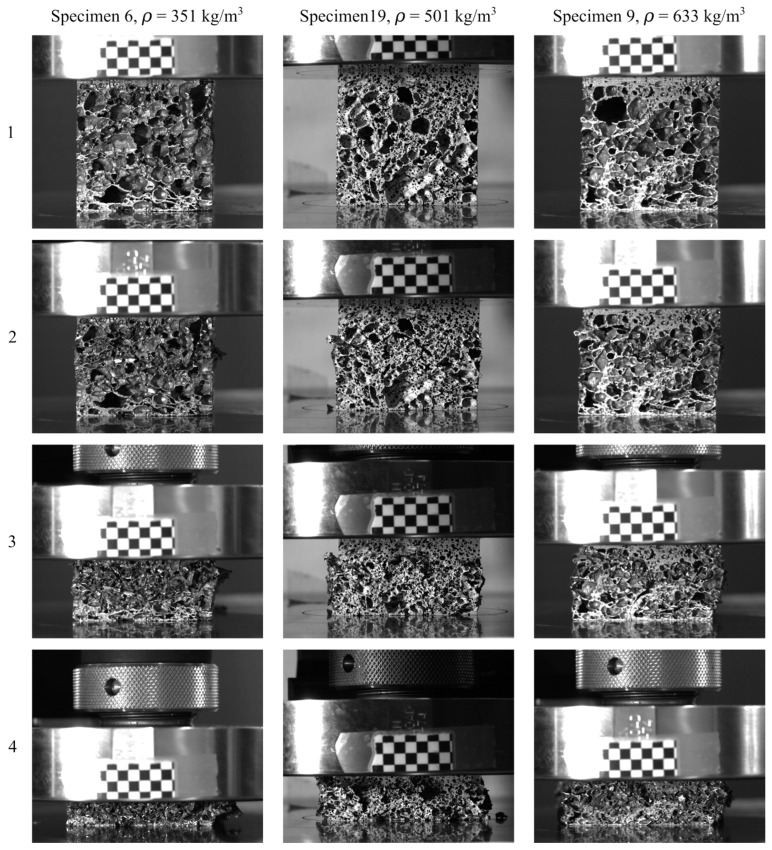
The deformation and fracture process of aluminium foam specimens during quasi-static compression from the tests of specimen 6 with ρ = 351 kg/m^3^, specimen 19 with ρ = 501 kg/m^3^, and specimen 9 with ρ = 633 kg/m^3^. The numbers 1 to 4 correspond to the numbers 1 to 4 in Figure 5.

**Figure 5:**
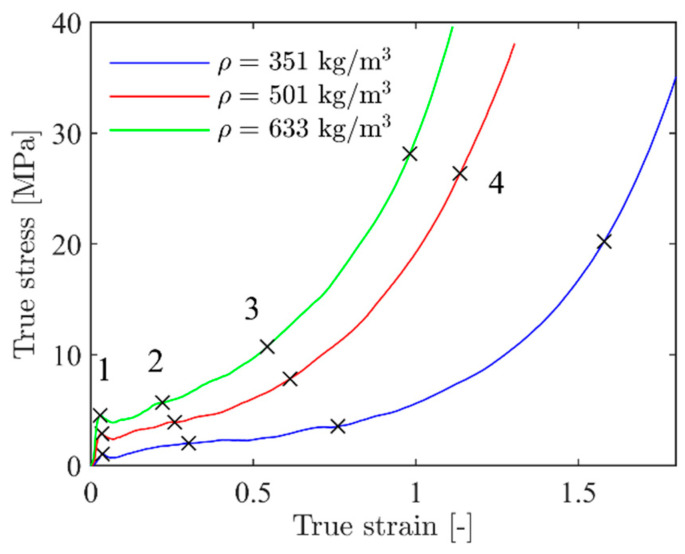
True stress and strain curves from the specimens/tests shown in Figure 4.

**Figure 6:**
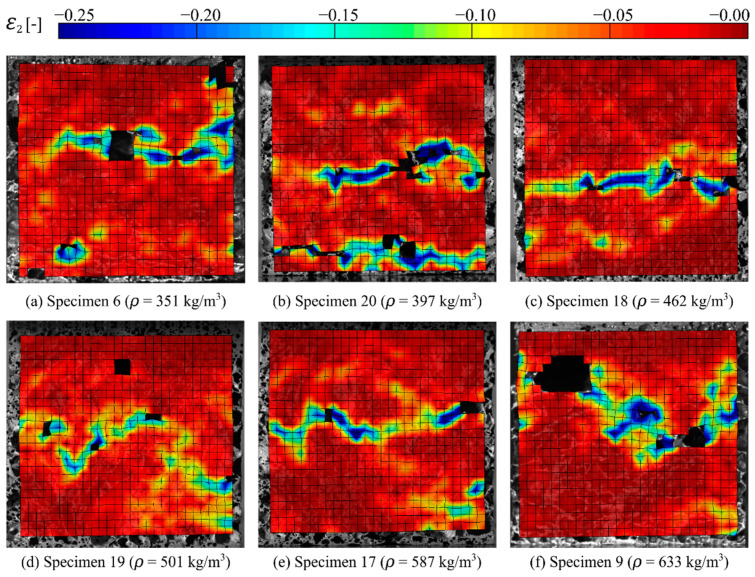
Measured surface strains in the thickness direction based on 2D-DIC at approximately 2 mm global deformation during quasi-static compression of the specimens shown in Figure 2.

Images of the deformation and fracture process of representative aluminium foam specimens during quasi-static compression tests are shown in Figure 4. Here, specimen 6 has the lowest density (ρ = 351 kg/m^3^), specimen 9 has the highest density (ρ = 633 kg/m^3^), while specimen 19 has a density (ρ = 501 kg/m^3^) close to the average density of 491 kg/m^3^ of the specimens tested. Figure 5 relates the images in Figure 4 to the measured true stress and true strain. Even though the density of the specimens in the figure is significantly different, the material shows similar behaviour during compaction. Since the voids just collapse without considerable material fracture, the deformation perpendicular to the direction of loading during plastic straining is negligible. Thus, it seems safe to state that the Poisson’s ratio during plastic deformation, $\nu^{p}$, is close to zero. Figure 6 provides measured strain fields in the thickness direction at approximately 2 mm global deformation during quasi-static compression of the specimens shown in Figure 2 based on 2D-DIC. Due to the crushing of the foam material, it was necessary to erode elements in the DIC-mesh to avoid premature termination of the DIC analyses. This was done when the major principal strain component $\varepsilon_{1}$ (in tension) reached a value of 0.7, and eroded elements are seen as black spots in Figure 6. The figure also shows that the strains localise in clear, but rather irregular, bands close to the centre of the specimens (except in specimen 20 where two bands can be seen). The location of these bands confirms well with the density measurements based on computed tomography presented in Section 2.3 showing that the strains localise in the areas with the lowest material density.

### 2.3. X-ray Micro Computed Tomography

**Figure 7:**
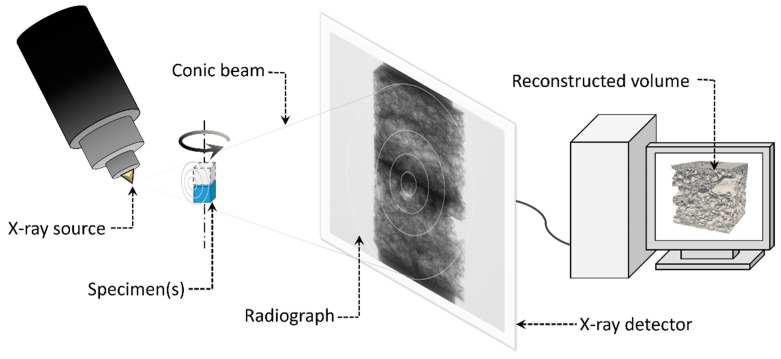
Principles and setup of the volume reconstruction using XRMCT.

X-ray micro computed tomography (XRMCT) was used to study and characterise the topology and morphology of the aluminium foam material. Five of the specimens (i.e., specimens 16–20) were studied using this method. A Nikon XT H225 ST MicroCT machine at the Norwegian University of Science and Technology (NTNU) was used. The machine is an X-ray tube-based device producing a conical beam of polychromatic X-rays. All scans presented herein were made with a wolfram reflection target, 142 kV/185 µA tube current, and a panel detector of 2000 × 2000 pixels, 200 μm pixel size, and 16-bit pixel depth. Each tomographic projection was collected using the average of two X-ray images with 1 s exposure time taken at 1571 azimuthal orientations and a total rotation angle of 360 degrees. The volume reconstruction was done using the commercial software Nikon CT Pro 3D (Version XT 3.1.3) and exported as an 8-bit depth grey-scale image stack. The spatial resolution of all scans was below 0.06 mm. The principles and setup of the volume reconstruction using XRMCT are illustrated in Figure 7.

**Figure 8:**
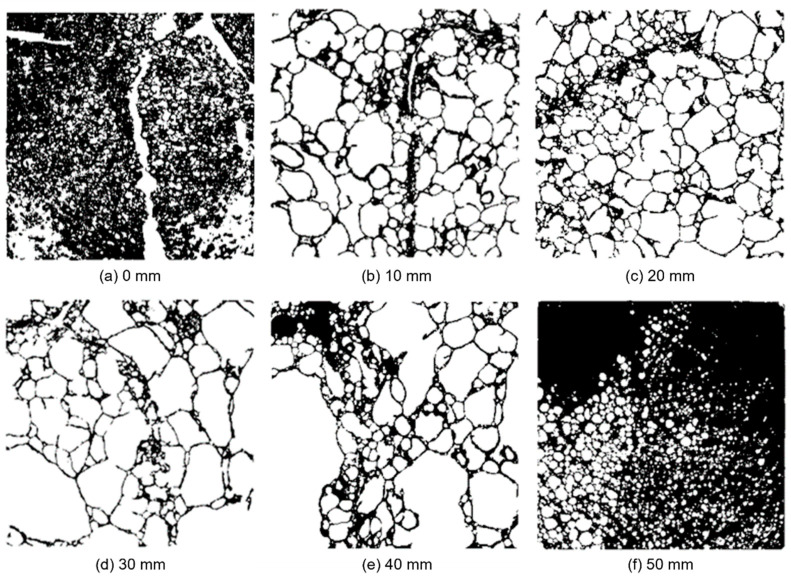
Binary image slices from XMRCT-data at six positions through the specimen’s thickness in the compression direction of specimen 19 with ρ = 501 kg/m^3^.

The raw image stack was post-processed using customised software routines made in Python 3.6. Each raw stack was filtered using a bilateral filtering scheme implemented in the OpenCV (version 4.0.0) module. Bilateral filters are both edge-preserving and noise-reducing, making them a good option for smoothing the grey-scale data while retaining the cellular structure at the same time. As a subsequent processing step, the filtered image stack was binarised using an implementation of Otsu’s method to find the optimal threshold grey-scale value to distinguish aluminium material from pores. Figure 8 presents binary image slices of specimen 19 with ρ = 501 kg/m^3^ at six positions through the specimen’s thickness in the compression direction. An obvious material inhomogeneity is seen both over the cross-section at each position and throughout the thickness. A rough check of the binary images from the CT scans indicated that the thickness of the cell walls was rather constant and independent of pore size in the centre part of the foam, but the cell wall thickness increased towards the external skins as the relative density increased significantly.

**Figure 9:**
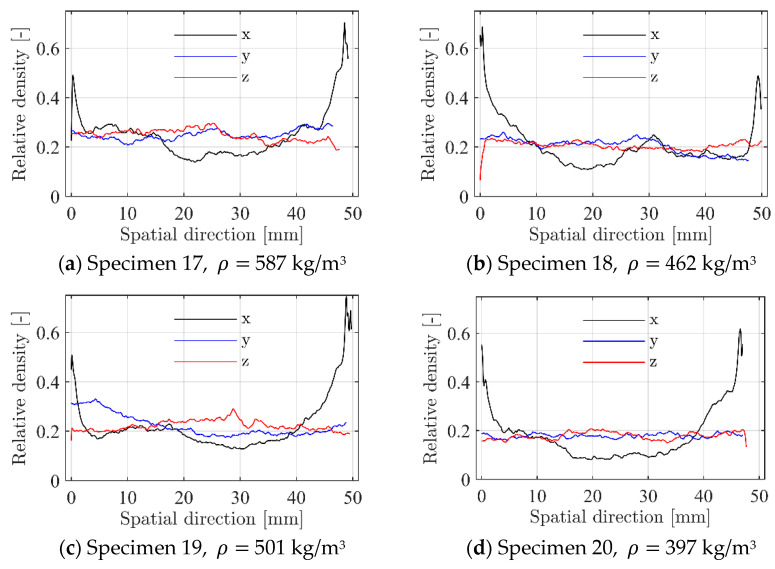
Variation in relative density across the specimens’ three spatial dimensions, where x is the thickness direction (including the external skins), y is the length direction, and z is the width direction.

The binary image stacks were used to study the inherent variation in the morphology of the specimens. A surface rendering of the binary stack of specimen 19 is shown to the right in Figure 7. Relative density is a key feature in characterising foams [8]. This feature was estimated based on the number of material pixels to total pixels in each binary image in the stack. Figure 9 presents the relative density variation across the specimens’ three spatial dimensions for four of the five CT-scanned specimens (specimen 17 is omitted due to brevity but revealed a similar variation). While the relative density of each individual specimen is seen to be rather constant and roughly 0.2 in the length (y) and width (z) directions, significant variations are seen across the specimens’ thickness (x) direction. Note further that the relative density reaches a local minimum of around 0.1 in the centre of the specimens, while it increases to above 0.5 towards the external skins. This strong heterogeneity in density over the thickness of the panel is the main reason for the strain localisation and plastic collapse in the centre of the specimens as seen in Figure 4 and Figure 6.

**Figure 10:**
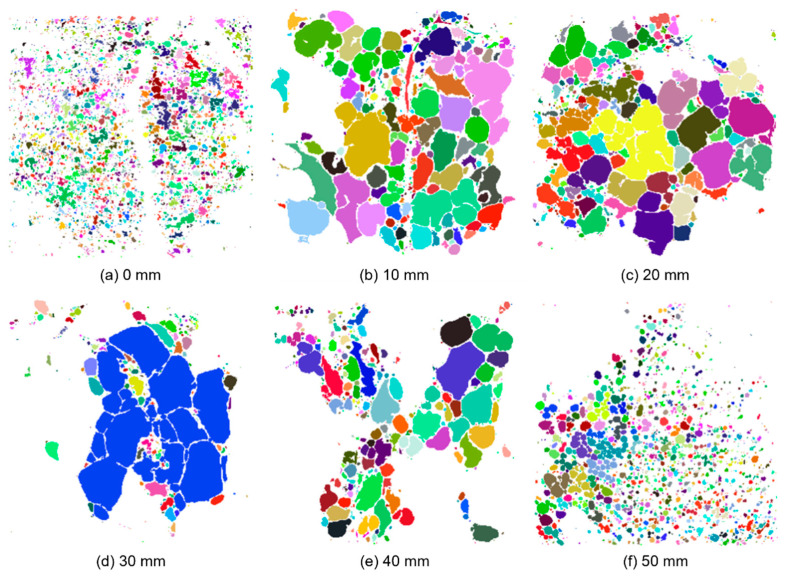
Pore morphological variation in the thickness direction of the aluminium foam material based on the binary image slices from Figure 8.

To further illustrate the pore morphological variation in the thickness direction of the aluminium foam, yet another post-processing step was applied to the binary image stack of specimen 19. All unconnected pores in the individual binary images were labelled, meaning that the pores were segmented from each other and given a unique integer label. The resulting image stack is referred to as the labelled image stack. This step was also achieved through processing using a custom-made Python routine applying functionality available in SciPy (version 1.2.0). Figure 10 presents the same cross-sections as in Figure 8, only with a unique colour given to each individual labelled pore. Note that all pores connected to the surrounding background are removed in the figures as these are not closed. The post-processing step illustrates an apparent difference in pore sizes across the studied cross-sections.

**Figure 11:**
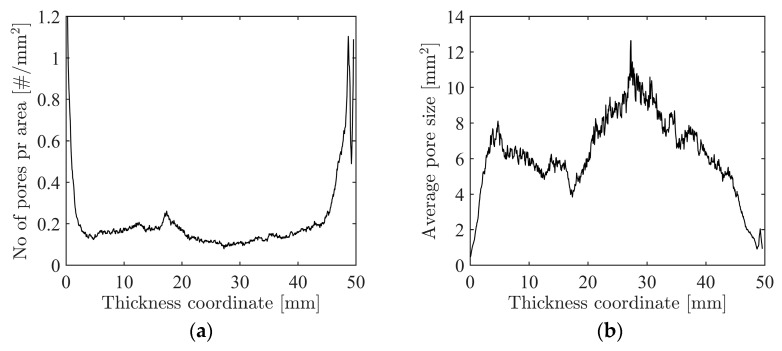
(**a**) Number (#) of pores (or clusters) per total pore area in each image slice, and (**b**) average pore sizes versus thickness coordinate.

The labelled image stack was further post-processed to quantify the inhomogeneity of the foam across the thickness, and Figure 11 presents (a) the number of pores per area in each image slice and (b) gives the average pore size. These plots illustrate that towards the edges of the specimen, there are many small pores, while towards the centre of the foam, there are much fewer and larger pores. This feature is also clearly seen in Figure 10. Even though such quantifications of the pore morphology and topology are too detailed for the analysis carried out in this study, it demonstrates the potential of using XRMCT data to numerically optimise the design and improve the behaviour of inhomogeneous foams with respect to energy absorption, thermal isolation, or similar properties.

## 3. Component Tests

### 3.1. Experimental Setup

The ballistic impact tests were performed in a compressed gas gun facility described in detail by Børvik et al. [45]. The main components of the gas gun are a 200-bar pressure tank, a firing unit for compressed gas, a 10 m long smooth barrel of calibre 50 mm, and a closed 16 m^3^ impact chamber. The gas gun is designed to launch a 250 g projectile/sabot package to a maximum velocity of 1000 m/s when helium is used as a propellant. In these tests, the sabot-mounted projectiles were fired at impact velocities just below and well above the ballistic limit of the aluminium foam panels by using compressed air. The 3D-printed serrated sabot separated immediately after leaving the barrel of the gas gun, and the sabot pieces were stopped in a sabot trap located 1.5 m behind the muzzle.

**Figure 12:**
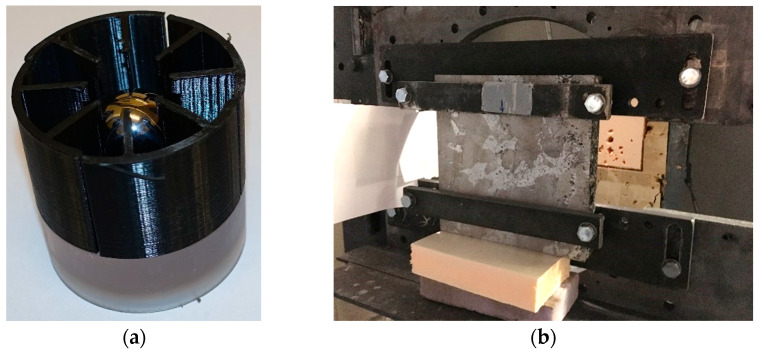
(**a**) Spherical projectile inserted in the 3D printed serrated sabot and (**b**) target panel inserted in the rigid test fixture.

The projectiles applied in these tests were ball bearing spheres made of steel. The spheres had a diameter of 20 mm, a mass of 32.64 g, and were assumed rigid during the penetration and perforation process of the much softer target. These projectiles were chosen in this study since they are believed to represent an idealised fragment generated by a blast load. A picture showing the projectile inserted in the five-pieced additive manufactured serrated sabot before testing is shown in Figure 12a. The 300 mm × 300 mm × 50 mm thick foam panel targets were placed in a rigid test fixture during testing (see Figure 12b). Two steel beams were attached to the test fixture by four M12 bolts that were used to clamp the top and bottom edge of the target; hence, the vertical sides of the panel were free with a span of approximately 180 mm. This setup has previously been successfully used in experimental studies on ballistic impact with different types of projectiles and target configurations (see, e.g., Dey et al. [46] and Holmen et al. [47]).

A Phantom v1610 high-speed camera with a recording rate of 50,000 fps was used to provide high-quality images of the penetration process from well before impact until complete perforation. The resulting images were used to investigate the perforation process and to measure the impact ($v_{i}$) and residual ($v_{r})$ velocities of the projectiles. Based on these measurements the ballistic limit curve and the ballistic limit velocity ($v_{\mathrm{bl}}$) were estimated by a generalised version of the Recht–Ipson model [48], given by

(3) $$v_{r}=a{\left(v_{i}^{p}-v_{\mathrm{bl}}^{p}\right)}^{1/p}\;\Rightarrow \;v_{\mathrm{bl}}={\left(v_{i}^{p}-{\left(\frac{v_{r}}{a}\right)}^{p}\right)}^{1/p}$$

where the model constants a, p, and $v_{\mathrm{bl}}$ were found from a best fit to the experimental results using the method of least squares.

### 3.2. Experimental Results

**Figure 13:**
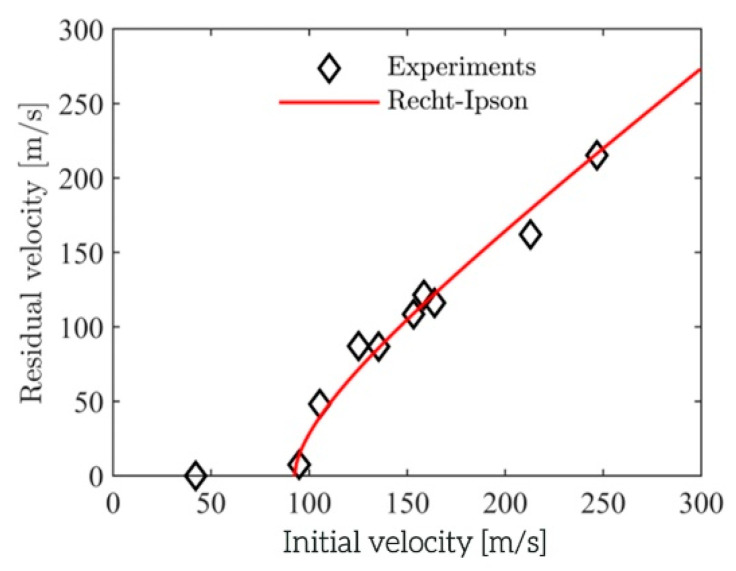
Ballistic limit curve compared to the experimental results from the ballistic impact tests on the aluminium foam panels.

In total, 10 ballistic impact tests on 10 individual aluminium foam panels were performed with the experimental setup described above. Based on the measured impact and residual velocities, the Recht–Ipson constants in Equation (3) were obtained from a best fit to the experimental results. The acquired constants were a=1, p=1.66, and $v_{\mathrm{bl}}=92.5$ m/s. The ballistic limit curve was constructed and is plotted in Figure 13 where it is compared to the experimental data. The agreement between the fitted ballistic limit curve and the experimental results is, in general, excellent, and the scatter in the test results is low.

**Figure 14:**
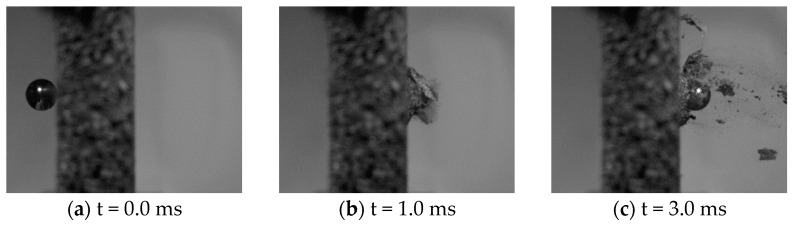
Typical images from the high-speed camera video of the perforation process at an impact velocity close to the ballistic limit velocity ($v_{i}=95\;m/s$ and $v_{r}=7.4\;m/s$).

**Figure 15:**
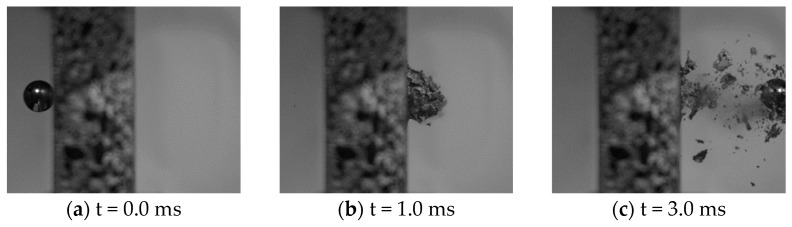
Typical images from the high-speed camera video of the perforation process at an impact velocity well above the ballistic limit velocity ($v_{i}=212\;m/s$ and $v_{r}=162\;m/s$).

**Figure 16:**
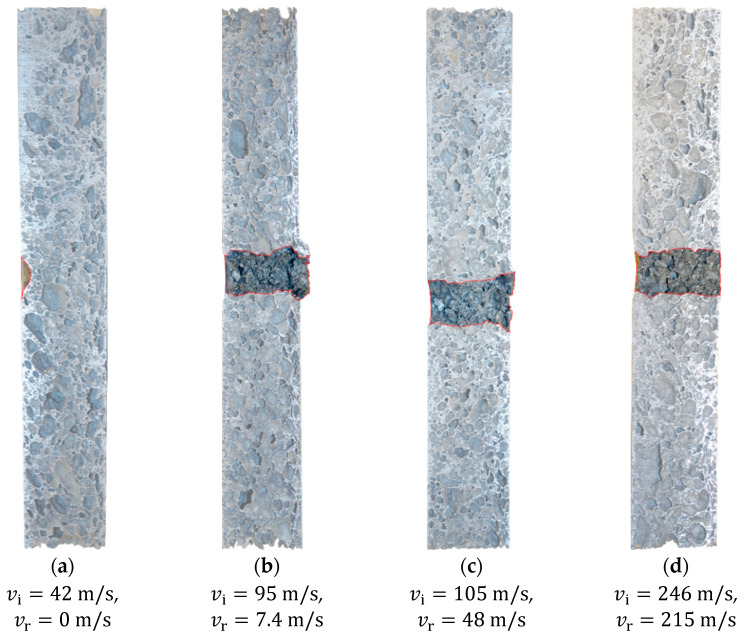
Slices of some of the aluminium foam panels after testing; the red lines highlight the penetration channel.

**Figure 17:**
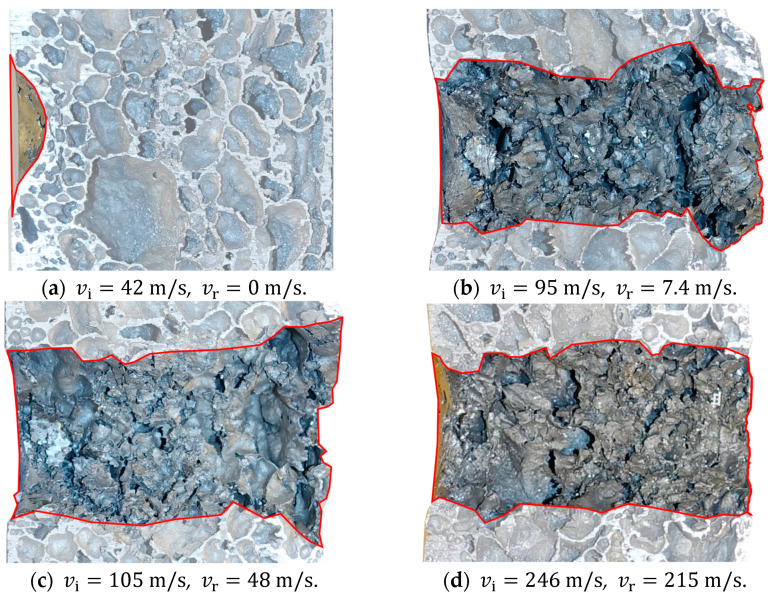
Close-up of the impact zone in the aluminium foam panels in Figure 16; the red lines highlight the penetration channel.

Figure 14 and Figure 15 show some typical images from the high-speed camera of the perforation process at impact velocities well above and close to the ballistic limit velocity of the aluminium foam panels, respectively. As seen, the projectile perforates the panel with limited fragmentation, and the perforation process looks similar and independent of the impact velocity. The perforation process takes less than 3 ms. Pictures of some sliced aluminium foam panels after testing are shown in Figure 16. For the lowest impact velocity, shown in Figure 16a, the projectile did not perforate the panel, and only a shallow indent at the surface is seen. Figure 16b–d shows panels from tests where complete perforation occurred. Even though both the impact and residual velocities differ significantly, the penetration channels look the same by visual inspection. The only noticeable difference is slightly more fragmentation from the rear side of the target plate at the highest impact velocities. A close-up of the penetration channels shown in Figure 16 is presented in Figure 17, where it is clearly seen that the cell structure of the foam is severely compressed and partly crushed. In addition, note the formed fragments seen on the rear side of the target plate in Figure 17b. These fragments are almost detached, since a vertical crack is clearly seen, but they have not been ejected from the surface of the plate.

## 4. Numerical Simulations

### 4.1. Constitutive Relation and Fracture Criteria

All numerical simulations presented in this work were conducted using the explicit finite element solver LS-DYNA [49]. The main objectives of the numerical work were to assess the level of accuracy obtainable in numerical simulations of ballistic impact on a highly inhomogeneous aluminium foam using a commercial solver and a standard material model and to investigate the effect and necessity of including detailed density measurements based on XRMCT data.

The loading conditions during ballistic events involve large plastic strains, high strain rates, and thermal softening due to self-heating in the affected materials. To account for these effects, a thermoelastic-thermoviscoplastic constitutive model such as the one proposed by Børvik et al. [50] should be applied to the material. However, Sun and Li [9] suggested that for the high strain-rate loading of aluminium foams, where an increase in temperature is to be expected, the effect of the thermal softening is much less significant than the strain-rate hardening, so possible thermal effects will be neglected in this study.

For the pressure-sensitive foam cores, the yield function should include a hydrostatic stress term in addition to the deviatoric stress term to account for the volumetric change when the cells of the material collapse under compression. The continuum-based isotropic constitutive relation for crushable foams proposed by Deshpande and Fleck [44] was adopted for this purpose. In this model, the equivalent stress $\sigma_{\mathrm{eq}}$ is given by

(4) $$\sigma_{\mathrm{eq}}^{2}\left(\sigma \right)=\sigma_{\mathrm{eq}}^{2}\left(\sigma^{\prime },\sigma_{H}\right)=\frac{1}{\left[1+{\left(\frac{\alpha }{3}\right)}^{2}\right]}\left[\sigma_{\mathrm{vM}}^{2}+\alpha^{2}\sigma_{H}^{2}\right]$$

where the von Mises equivalent stress $\sigma_{\mathrm{vM}}$ is defined by

(5) $$\sigma_{\mathrm{vM}}\left(\sigma \right)=\sigma_{\mathrm{vM}}\left(\sigma^{\prime }\right)=\sqrt{3J_{2}}=\sqrt{\frac{3}{2}\sigma^{\prime }:\sigma^{\prime }}$$

and $\sigma_{H}=\frac{1}{3}\mathrm{tr}\left(\sigma \right)$ is the mean hydrostatic stress. The parameter α governs the shape of the yield surface and is defined as

(6) $$\alpha^{2}=\frac{3G}{K}=\frac{9\left(1-2\nu^{p}\right)}{2\left(1+\nu^{p}\right)}$$

where K and G are the bulk- and shear modulus, respectively, and $\nu^{p}$ is the plastic coefficient of contraction. It is seen from Equation (6) that when $\nu^{p}=0.5$, as for pressure insensitive materials, $\alpha^{2}=0$, and the equivalent stress $\sigma_{\mathrm{eq}}\left(\sigma \right)$ in Equation (4) reduces to the von Mises equivalent stress $\sigma_{\mathrm{vM}}$.

The plastic rate-of-deformation tensor, $D^{p}$ is defined by the associated flow rule, viz.

(7) $$D^{p}=\dot{p}\frac{\partial f}{\partial \sigma }$$

where $\dot{p}$ is the equivalent plastic strain rate. The yield function is adopted in the form

(8) $$f\left(\sigma ,R\right)=\sigma_{\mathrm{eq}}\left(\sigma^{\prime },\sigma_{H}\right)-\sigma_{Y}\left(R\right)$$

The plastic rate-of-deformation tensor for the pressure-sensitive material is decomposed into a deviatoric and a hydrostatic part, viz.

(9) $$D^{p}=\dot{p}\frac{\partial f}{\partial \sigma }=\dot{p}\frac{\partial f}{\partial \sigma_{\mathrm{vM}}}\frac{\partial \sigma_{\mathrm{vM}}}{\partial \sigma }+\dot{p}\frac{\partial f}{\partial \sigma_{H}}\frac{\partial \sigma_{H}}{\partial \sigma }={\dot{\varepsilon }}_{\mathrm{vM}}n+\frac{1}{3}{\dot{\varepsilon }}_{v}I$$

where the von Mises equivalent plastic strain rate ${\dot{\varepsilon }}_{\mathrm{vM}}$ and the volumetric plastic strain rate ${\dot{\varepsilon }}_{v}$ are defined as

(10) $${\dot{\varepsilon }}_{\mathrm{vM}}=\frac{\dot{p}}{1+{\left(\frac{\alpha }{3}\right)}^{2}}\frac{\sigma_{\mathrm{vM}}}{\sigma_{\mathrm{eq}}},\;{\dot{\varepsilon }}_{v}=\frac{\alpha^{2}\dot{p}}{1+{\left(\frac{\alpha }{3}\right)}^{2}}\frac{\sigma_{H}}{\sigma_{\mathrm{eq}}}$$

By combining the equations above, it is possible to express the equivalent plastic strain rate explicitly in terms of ${\dot{\varepsilon }}_{\mathrm{vM}}$ and ${\dot{\varepsilon }}_{v}$, viz.

(11) $$\dot{p}=\sqrt{\left[1+{\left(\frac{\alpha }{3}\right)}^{2}\right]\left({\dot{\varepsilon }}_{\mathrm{vM}}^{2}+\frac{1}{\alpha^{2}}\;{\dot{\varepsilon }}_{v}\right)}$$

Based on the work of Hanssen et al. [51] and Reyes et al. [52], the flow stress is taken as

(12) $$\sigma_{Y}\left(R\right)=\sigma_{P}+R\left(p\right)=\sigma_{P}+\gamma \frac{p}{\varepsilon_{D}}+\alpha_{2}\ln\left(\frac{1}{1-{\left(\frac{p}{\varepsilon_{D}}\right)}^{\beta }}\right)$$

where $\sigma_{P}$ is the plateau stress, $\varepsilon_{D}$ is the true densification strain, and γ, $\alpha_{2},$ and β are material parameters governing the work hardening. This constitutive relation was implemented in LS-DYNA as material model 154 (*MAT_DESHPANDE_FLECK_FOAM) by Reyes et al. [52].

Hanssen et al. [51] proposed a relation where the material parameters are expressed in terms of the relative density by

(13) $$\left\{\sigma_{P},\alpha_{2},\gamma ,\frac{1}{\beta }\right\}=C_{0}+C_{1}{\left(\frac{\rho_{f}}{\rho_{f0}}\right)}^{n}$$

Here, $C_{0},C_{1},$ and n are constants, while $\rho_{f}$ and $\rho_{f0}$ are the density of the foam and the base material, respectively. In the special case of $\nu^{p}=0$ and uniaxial compression, the true densification strain can be expressed as

(14) $$\varepsilon_{D}=-\ln\left(\frac{\rho_{f}}{\rho_{f0}}\right)$$

As the material studied is assumed to have a plastic contraction of $\nu^{p}=0$, and the calibration tests are performed in uniaxial compression, the relations in Equations (13) and (14) were used to account for the density variation

It is difficult to establish a proper fracture criterion for metal foams, even though strain-, stress-, and energy-based criteria have been proposed in the literature (see, e.g., [52]). In this study, a simple stress-based fracture criterion is used, defined as

(15) $$\sigma_{1}\geq \sigma_{C}\;\Rightarrow \;\mathrm{Fracture}$$

to erode elements in the numerical models when the first principal stress $\sigma_{1}$ reaches a critical, positive value. Note that the first principal stress is zero in compression and positive in tension. Thus, a fracture can only occur under tensile loading. It is well known that more advanced fracture criteria for aluminium foams are available. However, since the main goal of this work was to investigate the behaviour of a highly inhomogeneous aluminium foam during impact loading, and not to examine details in the failure process, simulations involving more sophisticated fracture criteria are left for future studies.

In the following, material model 154 in LS-DYNA will be calibrated and used in three different ways. First, the foam is treated as homogeneous, and the model is directly calibrated based on the specimens’ measured true stress-strain response. Second, to include the inherent inhomogeneity in the foam’s density, the material constants are calibrated by taking the relative density into account through Equations (13) and (14). Third, the data from XRMCT will be directly used in the calibration of the material constants as the measured relative density in Equations (13) and (14). Finally, numerical simulations of the ballistic impact tests will be conducted using the first and third calibration of the model. Results from simulations based on the second calibration are left out due to brevity.

### 4.2. Homogeneous Foam Model

Hardening constants for representative foam specimens are given in Table 2. These were obtained by a direct fitting of Equation (12) to some of the experimental true stress-strain curves shown in Figure 3b using the method of least squares. The densification strain, $\varepsilon_{D}$, was determined from Equation (14) based on the measured overall density for each specimen.

**Figure 18:**
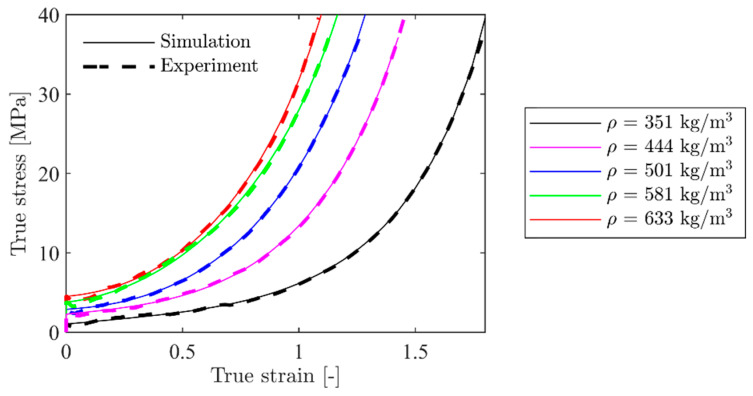
Comparison between simulated and experimental stress-strain curves for selected specimens with the homogeneous model.

The hardening parameters in Table 2 were validated by applying them in numerical simulations with material model 154 (Section 4.1) of the compression tests in LS-DYNA. In the numerical model, only a quarter of the cube, measuring 50 mm × 25 mm × 25 mm, was modelled. Thus, the two symmetry planes perpendicular to the axis of compression were utilised. 3D hexahedral brick elements with a size of 1 mm × 1 mm × 1 mm and a fully integrated S/R solid intended for elements with poor aspect ratio were applied as element type-2 in LS-DYNA. Note that this element size and type are unnecessarily expensive for these simulations, but they were used to keep the element size constant in all models. One side of the cube was compressed with nodal displacements, while the other side was fixed in all degrees of freedom. Numerical results for the five representative tests in Table 2 are shown in Figure 18. It is seen that the numerical model with the calibrated hardening parameters in Table 2 can predict the experimental true stress-strain curves accurately.

**Figure 19:**
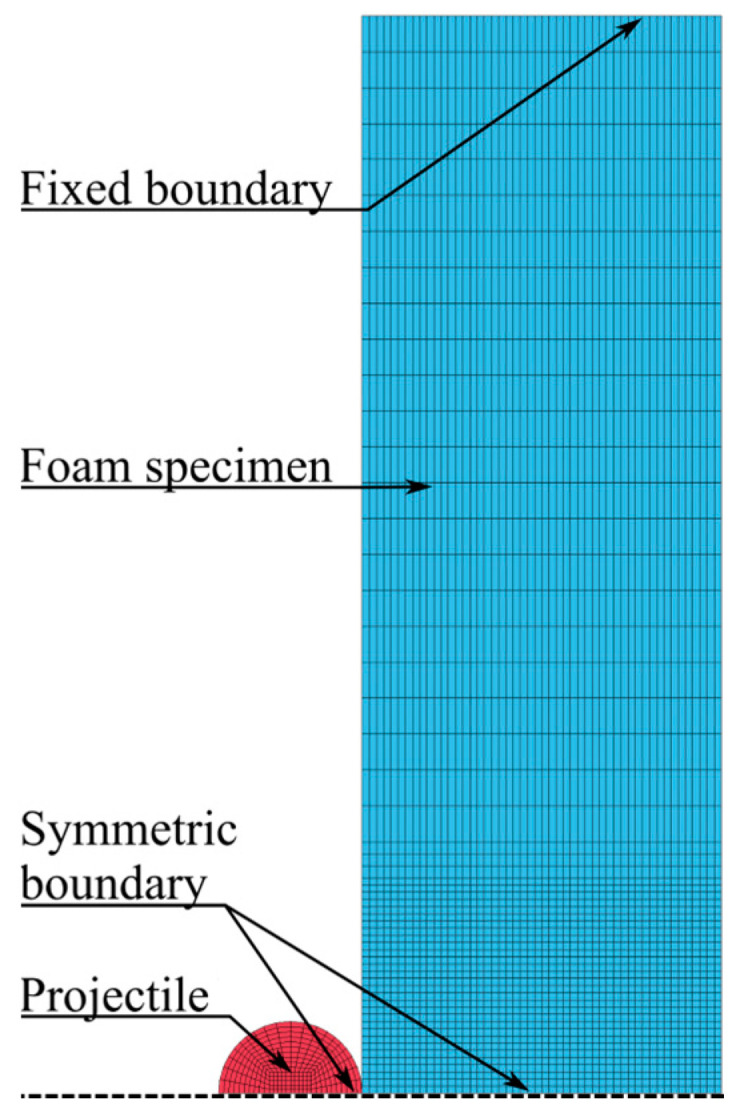
Numerical model of the ballistic impact tests with a homogeneous foam model.

The numerical model used to simulate the ballistic impact tests is shown in Figure 19. In the central impact zone of this model, eight-node hexahedral elements with size 1 mm × 1 mm × 1 mm and element formulation −2 were applied. Two symmetry planes were used to reduce the computational cost and an eroding surface-to-surface contact with a friction coefficient of 0.1 was used between the projectile and foam specimen. Due to the difference in elastic moduli between the projectile and foam, the soft option 1 for the contact was applied. With this option, the interface stiffness is based on nodal mass and the global time step size, resulting in a higher stiffness than the standard method using the bulk modulus. This method is preferred when soft materials, such as foams, interact with much stiffer materials, for example, metals. Symmetric boundary conditions were used at the symmetry planes, while the foam specimen was fixed at the outer boundary. The projectile was modelled as a discrete rigid solid with elastic material properties for steel, i.e., E=210 GPa, ν=0.3, and $\rho =7850\;\mathrm{kg}/m^{3}$. The projectile was given various initial velocities normal to the target plate with a nodal boundary condition. Symmetric boundary conditions were applied to the projectile at the symmetry planes.

As before, the foam material was modelled with material model 154 in LS-DYNA with the hardening constants in Table 2. Material failure was included with MAT_ADD_EROSION based on the relation in Equation (15), and failure occurred when the first principal stress exceeded a critical value. In the model, the element is eroded when all integration points in an element exceed the critical stress. Element erosion is accomplished in LS-DYNA by setting all components in the stress tensor to zero. The mass is still contained in the connected nodes, while the internal energy is included in the energy balance. The critical stress $\sigma_{C}$ was related to the material plateau stress $\sigma_{p}$ by

(16) $$\sigma_{C}=c_{f}\times \sigma_{p}$$

where $c_{f}$ is a constant unique for each foam density.

**Figure 20:**
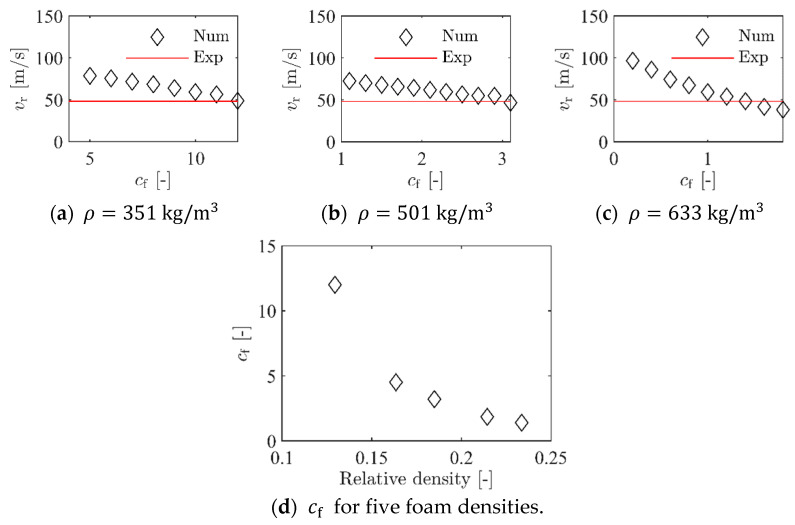
Numerical results for calibration of critical stress factor $c_{f}$.

The density in the central impact area was not exactly known for the plates but was assumed to be within the density range for the compression specimens. Thus, for calibration of the failure criterion, one ballistic impact test was simulated with the five foam densities given in Table 2. The ballistic impact tests with $v_{i}=105.4\;m/s$ and $v_{r}=48.2\;m/s$ were used for this purpose. For each of the five chosen foam densities, simulations were performed where $v_{i}$ was kept constant at 105.4 m/s and $c_{f}$ was varied until $v_{r}$ was the same as in the experiment. Figure 20a–c shows how changing $c_{f}$ affects $v_{r}$ in the simulations with three of the foam densities. There is a rather linear relationship between $c_{f}$ and $v_{r}$ for all densities that were used. For all five foam densities, a value for $c_{f}$ that gave the same $v_{r}$ as in the experiment was found. These values are given in Table 2. The value for $c_{f}$ was highly dependent on the density, as shown in Figure 20d.

**Figure 21:**
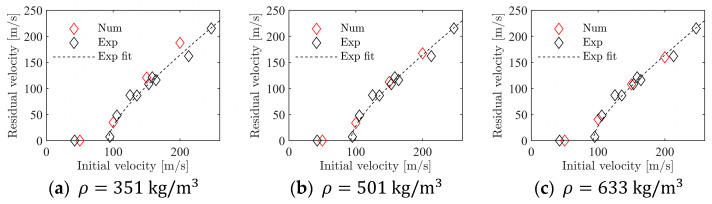
Validation of the numerical model for the penetration test with homogeneous foam model.

To validate the calibrated values for $c_{f}$, a series of four simulations of the ballistic impact tests was performed for each foam density. Here, $c_{f}$ was kept constant at the values given in Table 2, while $v_{i}$ was varied from 50 m/s to 200 m/s in 50 m/s increments, such that the entire experimental range was covered. Figure 21a–c shows the outcome from these simulations where they are compared to the experimental results. For all three foam densities, the numerical results are close to the experimental data, even though a small deviation among the numerical results is seen at the highest impact velocity. In any case, it seems safe to state that the numerical model with homogeneous foam density can accurately predict the experimental results from the ballistic impact tests.

### 4.3. Inhomogeneous Foam Model

**Figure 22:**
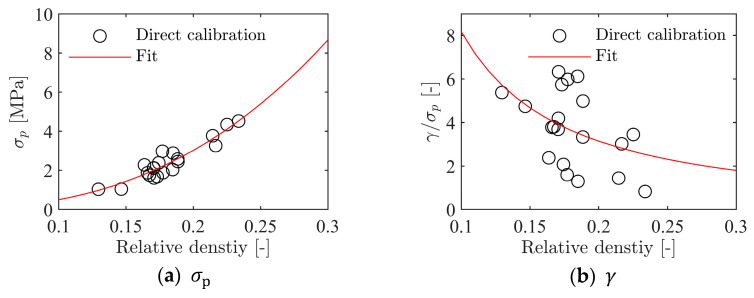

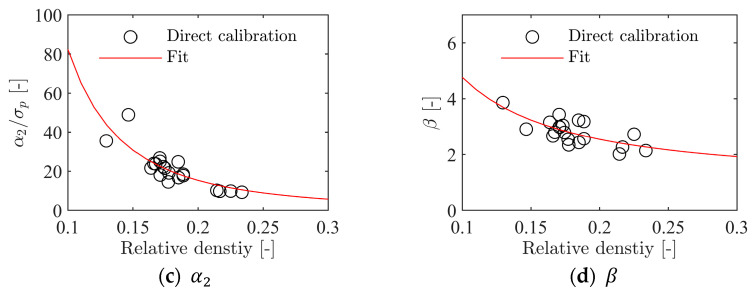
Calibration of hardening constants to relative density.

To model the inhomogeneity in the density of the foam found both by visual inspection, direct measurements, and XRMCT, the material constants had to be correlated with the foam density. Here, Equation (13) was used to relate the hardening constants found in the previous section to the specimen density. Note that data from all 20 uniaxial compression tests were used in this calibration. For each of the hardening constants, $\sigma_{P},\alpha_{2},\gamma ,$ and β, the constants in Equation (13) were found by least-squares fits to the calibrated model data; their values are given in Table 3. Figure 22 shows the correlation between the fitted curves based on Equation (13) and the calibrated hardening constants.

**Figure 23:**
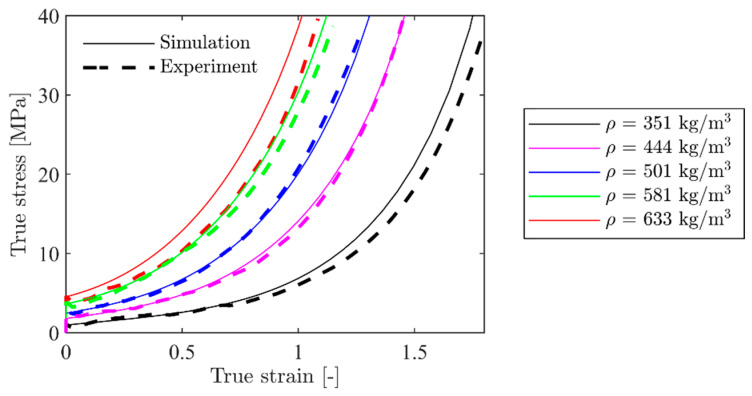
Comparison between numerical and experimental results for the homogeneous model with hardening constants from Table 3.

The density-dependent hardening constants in Table 3 were validated with the homogeneous numerical model of the compression test described in the previous section. In this case, the hardening constants for each model, i.e., $\sigma_{p},\;\gamma ,\;\alpha_{2},$ and β, were generated with the constants in Table 3 and the respective relative density for each specimen inserted in Equation (13). The resulting true stress-strain curves from these simulations are presented in Figure 23. These do not correlate as well with the experimental results as the directly calibrated homogeneous model shown in Figure 18. The results fit well for the intermediate densities, but not as well for the lowest and highest density. The reason for the deviation at the extremities is the lack of data in these regions of the range of measured densities for the material specimens. However, the overall correlation between the simulations and experiments is satisfactory for the density-dependent calibration.

**Figure 24:**
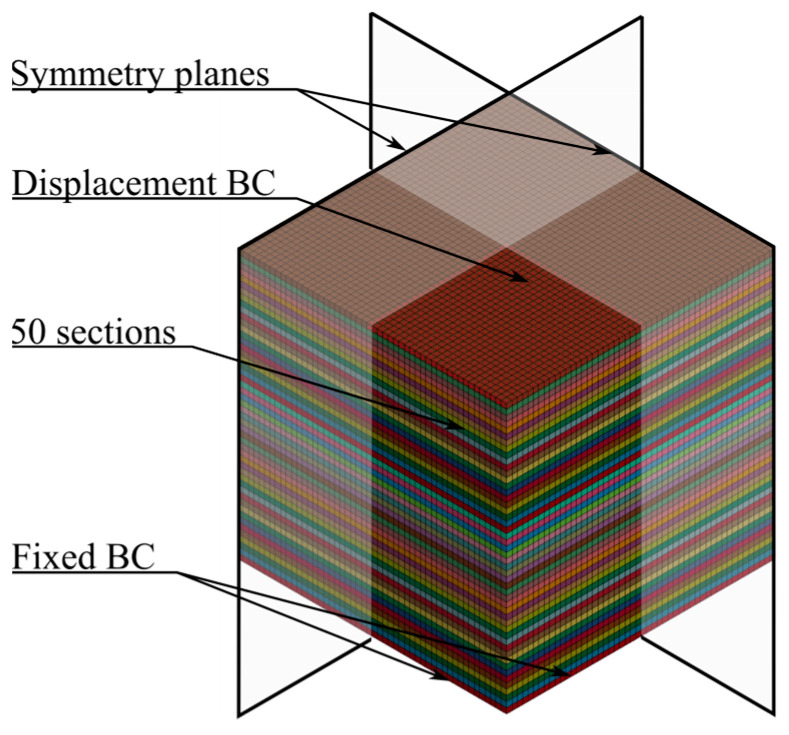
Numerical model with 50 sections of the compression tests with XRMCT density data.

**Figure 25:**
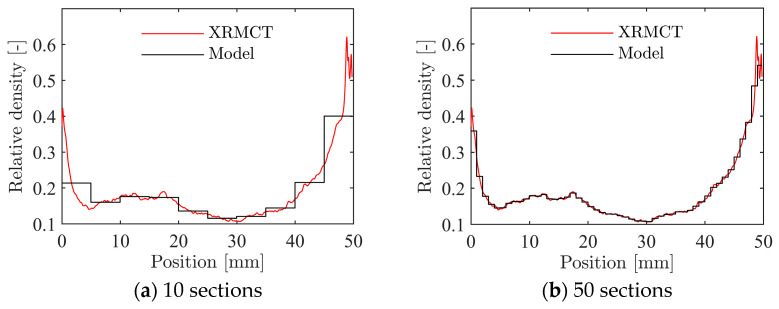
Discretisation of XRMCT data of specimen 19, $\rho =501\;\mathrm{kg}/m^{3}$ for application in the inhomogeneous numerical model.

To establish Figure 22, the foam itself was modelled as homogeneous but with density-dependent constants in the material model. As found from the XRMCT scans in Section 2.3, the density varies significantly in the through-thickness direction of the foam. In the following, the XRMCT data will be directly used to model the inhomogeneity of the material. For the compression tests, a schematic of the numerical model is shown in Figure 24. Here, the numerical model described in Section 4.2 was split into a number of unique homogeneous parts across the height of the specimen. All nodes on the interface between the parts were merged to create a continuous model. For each part, the material constants were assigned with their respective relative density using Equations (13) and (14) based on XRMCT and the constants in Table 3. To investigate the effect of the discretisation of the homogeneous sections, simulations with a range from 50 × 1 mm to 1 × 50 mm thick sections were run. For each section, the measured relative density from XRMCT was averaged over the height, as shown for 10 and 50 sections in Figure 25a,b, respectively. In the following, all simulations were performed with XRMCT data for specimen 19, with an average density of $\rho =501\;\mathrm{kg}/m^{3}$.

**Figure 26:**
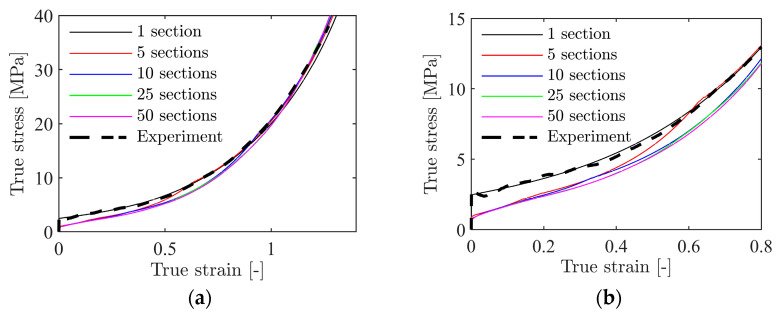
True stress-strain curves from simulations of a uniaxial compression test of a foam cube with a global density of $\rho =501\;\mathrm{kg}/m^{3}$, but different numbers of unique material layers, where (**a**) shows the true stress-strain curve until densification and (**b**) shows the yielding and hardening until a true strain of 0.8.

**Figure 27:**
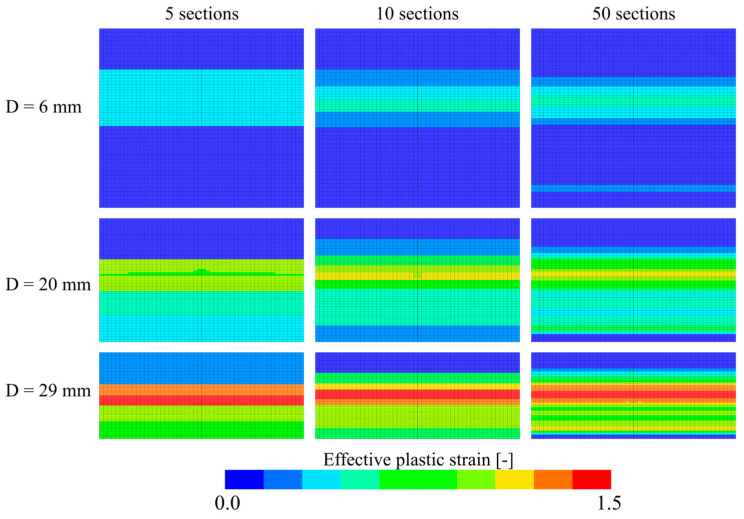
Plastic strain fields of the numerical models with 5, 10, and 50 unique material sections.

Figure 26a shows true stress-strain curves from simulations of the inhomogeneous foam model with 1 to 50 individual sections across the foam thickness. Overall, the models represent the experimental result well, especially at higher strains. There is a significant difference in the initial yield stress as the number of foam sections is increased. This is clearly seen in Figure 26b where the stress-strain response until a true strain of 0.8 is shown. The homogeneous (1 layer) model follows exactly the experimental curve. However, when inhomogeneity is introduced, using 5 to 50 layers, the stress is significantly reduced in this region. Figure 27 shows that the deformation localises in some sections using the inhomogeneous model, as is also seen in the experimental tests (Figure 6). This happens because the density is lower in these sections, and thus, the initial yield strength and hardening are lower. Because the material constants were calibrated based on the global compressive response of the specimens, the strength in the low-density central sections is underestimated and the numerical results deviate somewhat from the experimental results. Note also that the simulated strain fields shown in Figure 27 look more similar to the experimental results in Figure 6 as the number of sections is increased. For the most refined model with 50 × 1 mm thick sections, two bands appear where the material yields at low global compressive strains. In conclusion, the inhomogeneous model can capture the localisation behaviour of the foam found experimentally, but with a somewhat reduced accuracy of the stress-strain response.

**Figure 28:**
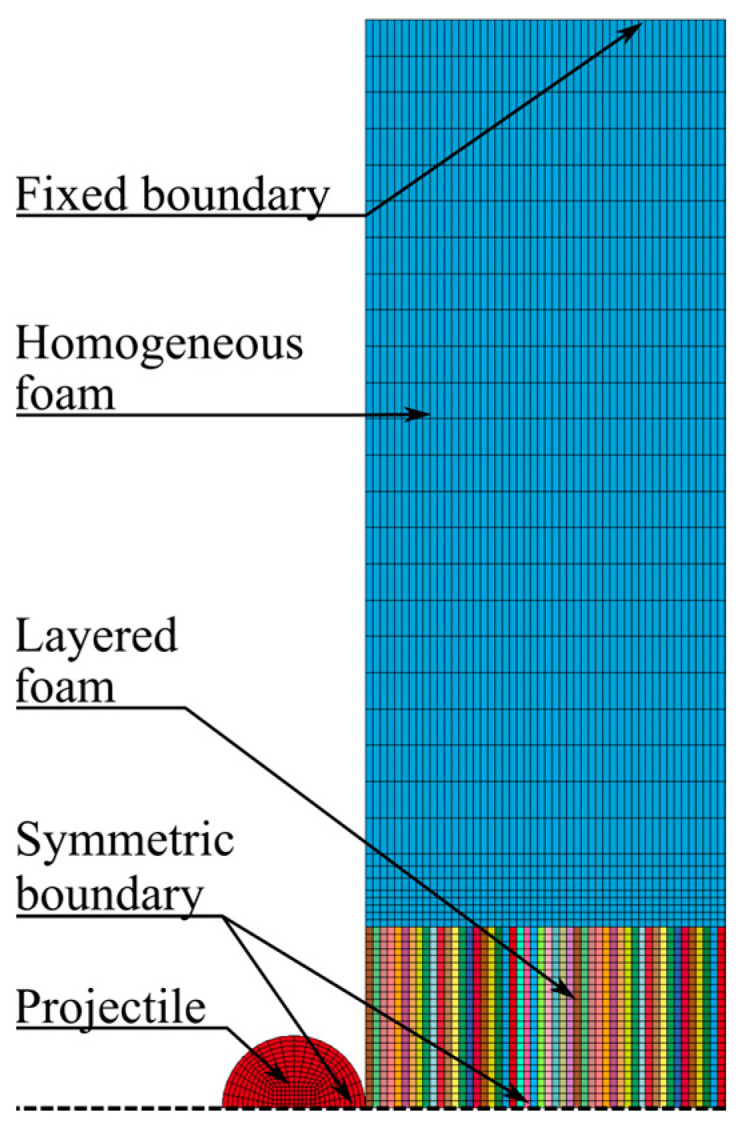
Numerical model of penetration tests with inhomogeneous foam.

**Figure 29:**
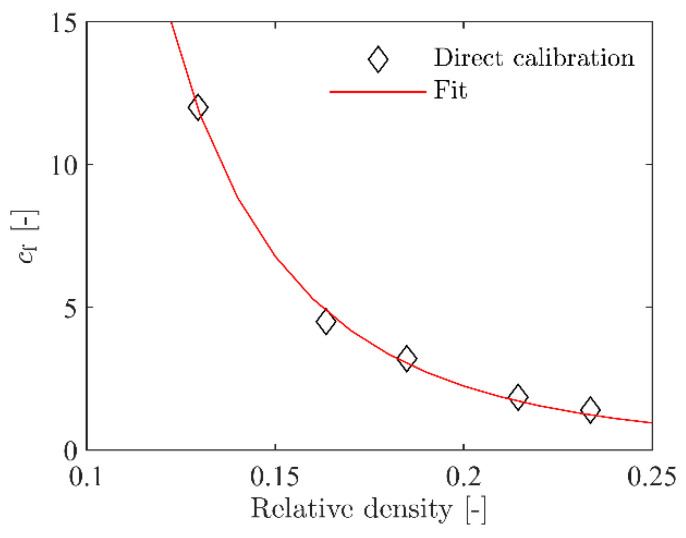
Calibration of density-dependent constants for the failure criterion.

The numerical model used for simulating the ballistic impact test with an inhomogeneous foam is shown in Figure 28. It is mainly the same model as used in the homogeneous model of the ballistic impact test, described in Section 4.2, but here the central impact area, measuring 50 mm × 50 mm × 50 mm, was replaced with a section of 50 × 1 mm thick parts. As in the simulations of the compression test, this makes it possible to distribute the density-dependent material parameters based on the XRMCT data within the impact zone. Outside the central impact zone, the foam was modelled as homogeneous with the average density measured with XRMCT. To relate the failure criterion to the foam density, Equation (13) was used to fit the failure stress factor $c_{f}$ in Equation (16) to the results obtained with the homogeneous model in Section 4.2. The obtained constants for Equation (13) are given in Table 3, while a comparison between the directly calibrated $c_{f}$ and the fitted parameters is shown in Figure 29. In all the simulations of the ballistic impact test with the inhomogeneous foam model, the measured densities of material specimen 19 with an average density of $\rho =501\;\mathrm{kg}/m^{3}$ was used, i.e., the density measured by XRMCT for the central impact area and the average density for the surrounding area.

**Figure 30:**
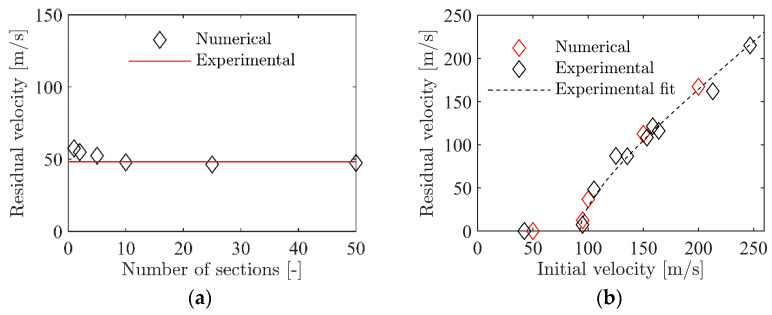
Numerical results for the inhomogeneous model of the ballistic impact tests: (**a**) the residual velocity of the projectile as a function of the number of unique foam sections and (**b**) comparison of the model results with 50 sections to the experimental results.

To validate the inhomogeneous modelling of the ballistic impact tests, a series of simulations with 1 to 50 unique foam sections was performed. As with the homogeneous model in Section 4.2, the initial velocity $v_{i}$ was 105.4 m/s in all simulations. The resulting residual velocities $v_{r}$ with different numbers of foam sections are shown in Figure 30a. The experimental result, $v_{r}=48.2$ m/s, is shown as the red line in the figure. With only one foam section, i.e., a homogeneous model, the residual velocity of $v_{r}=57.4\;m/s$ is slightly higher than the experimental value. From the direct calibration of the failure criterion in Section 4.2, it was found that the residual velocity in the simulations is highly sensitive to changes in the value of $c_{f}$. Thus, the small difference in $c_{f}$ introduced using a fitted curve results in this variation. As the number of unique material sections is increased, $v_{r}$ in the simulations is reduced. For 10, 25, and 50 section, $v_{r}$ is within 1–2 m/s of the experimental result. The model with 50 unique foam sections was then used to validate the inhomogeneous modelling of the ballistic impact test. As in the homogeneous model, this was done by a series of simulations with $v_{i}$ ranging from 50 m/s to 200 m/s in 50 m/s intervals. A comparison of these numerical results and the experimental results is given in Figure 30b. As seen, the numerical results give a good representation of the experimental results and the numerical results are well within the experimental scatter. Note also that the quantitative results in terms of residual velocity are very similar to the homogeneous model presented in Figure 21b.

**Figure 31:**
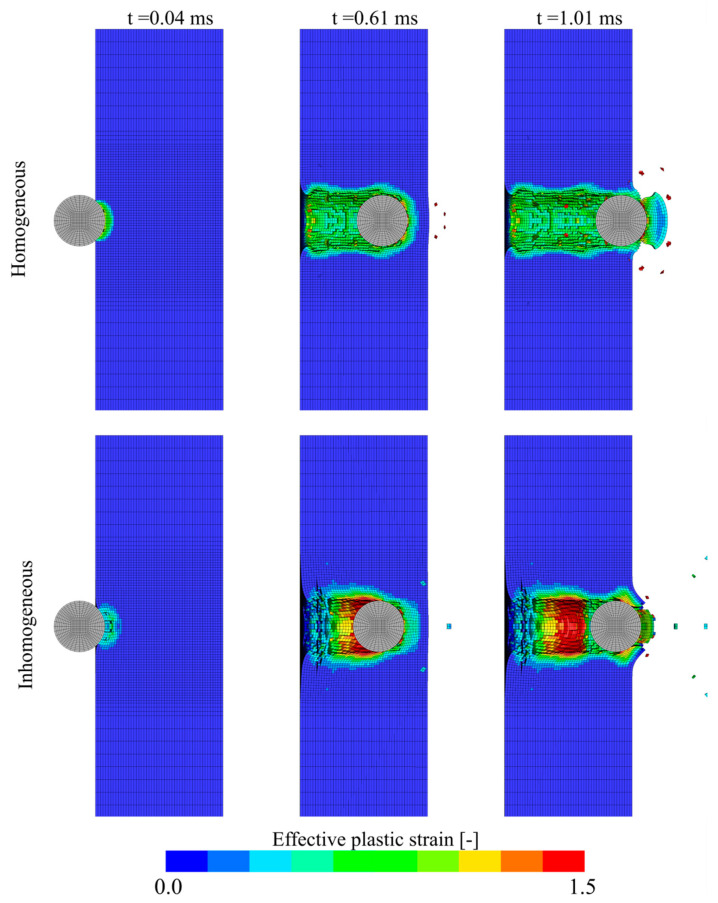
Timelapse of homogeneous and inhomogeneous ballistic impact models at $v_{i}=95\;m/s$.

Finally, the ballistic impact test with an initial velocity $v_{i}=95\;m/s$ was simulated both with the homogeneous and the inhomogeneous model. In this test, the residual velocity was only 7.4 m/s, i.e., very close to the calculated ballistic limit. As both modelling strategies gave comparable results for the entire range of impact velocities used experimentally, this limit case was used to differentiate the modelling techniques. At an impact velocity of 95 m/s, the residual velocity was found to be 24.9 m/s and 12.5 m/s for the homogeneous model and the inhomogeneous model, respectively. Considering the complexity of the foam material in this study, both models perform very well for this case, but the inhomogeneous model is closer to the experimental result than the inhomogeneous model. This may be a coincidence as the density of the foam panels used in the ballistic tests was not explicitly measured, and the numerical results are solely based on the measured average density and XRMCT data from material specimen 19. However, the timelapses of the ballistic impact simulations, shown in Figure 31, may explain why the inhomogeneous model is closer to the experiment result in this particular case. Initially, for the first 0.6 ms, both models give similar responses. However, for the last part of the penetration process, when the projectile is about to perforate the foam panel, the higher density in the outer layer of the inhomogeneous model distributes the plastic deformations away from the exit hole. This process dissipates energy and slows down the projectile more for the inhomogeneous model than for the homogeneous model. The size of the ejected plug and the deformation around the exit hole are also more similar to the experiment for the inhomogeneous than for the homogeneous model.

## 5. Concluding Remarks

In this study, the ballistic impact response of a highly inhomogeneous aluminium foam panel produced by the powder compaction method has been investigated both experimentally and numerically. First, to reveal the mechanical response of the material, a material test program involving 20 uniaxial compression tests on cubic specimens loaded in the thickness direction of the foam was carried out, and a significant difference in both density and the stress-strain response was obtained among the different samples. This dispersion was related to the highly inhomogeneous and complex cell structure of the foam caused by the production process. Measurements based on two-dimensional digital image correlation confirmed that the strain localised in distinct bands close to the centre of the specimen during compaction. To further characterise the pore topology and morphology, several specimens were scanned using X-ray micro computed tomography (XRMCT). Based on the post-processing of the image stacks, the variation in relative density across the specimens’ spatial directions was measured and the volumes were reconstructed. From these measurements, it was readily seen that while the relative density of the foam was almost constant in the in-plane directions, significant variations with a local minimum in the vicinity of the centre of the specimens’ thickness direction were observed. XRMCT data was also used to extract some pore statistics, such as the number of pores and average pore size in different directions, which are vital information if the goal is to optimise the foam design with respect to energy absorption or thermal isolation. Post-processing of the XRMCT data turned out to be a very powerful tool to increase our understanding of the behaviour of a highly inhomogeneous porous material exposed to impact loading.

In the second part of the study, ballistic impact tests were conducted using a compressed gas gun facility where rigid spheres were launched toward aluminium foam panels at a velocity range between 50 and 250 m/s. Based on the initial and residual velocities measured from the high-speed camera, the ballistic limit velocity and ballistic limit curve were constructed. This was done by a best fit using the method of least squares to the generalised Recht–Ipson model. Excellent agreement between the fitted curve and the experimental data was obtained, and the scatter in results was low. The high-speed camera also provided high-quality images of the penetration and perforation process and revealed that the perforation process is similar and independent of the impact velocity.

Finally, numerical simulations of the ballistic impact problem were carried out using a crushable foam model available in the explicit FE solver LS-DYNA. The constitutive relation was calibrated in different ways, either by direct fits to the true stress-strain curves from material tests or by the use of density-dependent material constants and XRMCT data, while a simple stress-based fracture criterion was established to describe material failure. The fracture criterion was calibrated by inverse modelling of one of the ballistic impact tests. Such an approach was found necessary to have reliable fracture predictions of the complex material; it transpired that the fracture prediction was excellent at all impact velocities even though only one experimental test was used in the calibration. Very good agreement between the simulated and experimentally obtained ballistic limit velocity and curve was obtained independent of modelling strategy, even though the results based on XRMCT data were slightly closer to the experimental results. Thus, this study demonstrates that if the ballistic perforation resistance of a foam material used as the core in a sandwich structure is the main goal, a standard material calibration based on compression tests seems sufficient. If, on the other hand, the core material is to be optimised with respect to energy absorption or thermal isolation, XRMCT data are both required and extremely useful. The main findings from the work can be summarised as follows:

- Aluminium foams produced by the powder compaction method may have a highly inhomogeneous and complex pore structure with a strong variation in density distribution throughout the spatial dimensions that affect the mechanical properties of the material and causes a significant spread in the stress-strain response.
- Although complex, post-processing of XRMCT data can be used to accurately characterise the topology and morphology of the foam and provide pore statistics that can be used to understand and optimise the behaviour of the material.
- The ballistic perforation resistance of the aluminium foam can be readily determined in a gas gun facility. Even though the material was complex, the penetration and perforation process was similar with limited fragmentation independent of impact velocity, and the spread in the experimental results was low.
- Numerical simulations using a crushable foam model available in LS-DYNA gave excellent predictions of the ballistic limit velocity and curve, independent of whether the constitutive relation was directly calibrated based on material tests or XRMCT data.
- It was, however, found necessary to calibrate the fracture criterion by inverse modelling of one of the ballistic impact tests to have reliable fracture predictions.

## Figures and Tables

**Table 1:** Estimated mechanical properties of representative foam specimens.

Specimen #	Density *ρ* [kg/mm^3^]	Elastic Modulus *E* [GPa]	Yield Stress $\sigma_{y}$ [MPa]	Plateau Stress $\sigma_{p}$ [MPa]	Densification Strain $\varepsilon_{D}$ [-]
6	351	2.6	1.03	1.97	2.04
19	501	4.6	2.88	4.16	1.68
9	633	6.8	4.52	6.58	1.45

**Table 2:** Hardening constants for representative foam specimens.

Specimen #	Density ρ [kg/mm^3^]	$\sigma_{p}$ [MPa]	$\varepsilon_{D}$ [-]	γ [MPa]	$\alpha_{2}$ [MPa]	β [-]	$c_{f}$ [-]
6	351	1.03	2.04	2.04	2.04	3.86	12
12	443	2.27	1.81	5.41	1.81	3.15	4.5
19	501	2.88	1.68	1.45	1.68	2.43	3.2
2	581	3.77	1.54	5.45	1.54	2.01	1.85
9	633	4.16	1.45	1.68	1.45	2.13	1.4

**Table 3:** Density-dependent hardening and failure constants for the foam.

	$\sigma_{p}$ [MPa]	$\gamma /\sigma_{p}$ [-]	$\alpha_{2}/\sigma_{p}$ [-]	β [-]	$c_{f}$ [-]
$C_{0}$	0	0	0	1.02	0
$C_{1}$	198.4	0.34	0.31	0.19	0.0046
n	2.60	−1.37	−2.42	−1.29	−3.85

## Data Availability

The raw/processed data required to reproduce these findings of this study may be made available from the corresponding author upon request.
